# Effect of treadmill exercise on skeletal muscle autophagy in rats with obesity induced by a high-fat diet

**DOI:** 10.20463/jenb.2017.0013

**Published:** 2017-09-30

**Authors:** Do Keun Cho, Dong Hun Choi, Joon Yong Cho

**Affiliations:** 1. Laboratory of Exercise Biochemistry, Korea National Sport University, Seoul Republic of Korea

**Keywords:** High-fat diet, Autophagy, Treadmill exercise, Obesity

## Abstract

**[Purpose]:**

This study aimed to investigate the effects of treadmill exercise on body weight, blood biochemistry, and autophagy.

**[Methods]:**

Triglyceride, total cholesterol, low-density lipoprotein cholesterol, high-density lipoprotein cholesterol, insulin, and glucose levels were measured, the Homeostatic Model Assessment of Insulin Resistance (HOMA-IR) score was calculated, and the soleus muscle was analyzed for autophagy-related factors (Beclin-1, p62, LC3, Lamp-2) in rats with obesity induced by a high-fat diet. Eight-week-old Sprague Dawley rats were fed a high-fat diet for 35 weeks and then subjected to 10 weeks of treadmill exercise. The experimental group was divided into a Normal Diet-Sedentary (ND-SED, n=8) group, an (High-Fat Diet-Sedentary (HF-SED, n=8) group, and an High-Fat Diet + Treadmill Exercise (HF-TE, n=8) group. The intensity of treadmill exercise was set as 8 m/min for 5 min, 11 m/min for 5 min, 15 m/min for 20 min, and 11 m/min for the last 5 minutes. A glucose tolerance test was performed at the 2nd and 8th week of exercise by sampling of tail blood.

**[Results]:**

With endurance exercise, the HFTE group showed a significant decrease in body weight, with improved blood biochemical indices and HOMA-IR scores, in comparison with the HF-SED group. However, there was no significant difference in Beclin-1, p62, LC3, and Lamp-2 proteins as measured by autophagic flux in the soleus muscle.

**[Conclusion]:**

Treadmill exercise induced improvements in body weight, body fat, and biochemical indicators of obesity and Type 2 diabetes, but had no effect on autophagy in soleus muscle.

## INTRODUCTION

Westernized eating habits and lack of exercise have increased the prevalence of various diseases. Among these, Type 2 diabetes mellitus (T2D) is characterized by the failure of normal glucose absorption and increased blood glucose levels^1^. At present, there are 400 million patients worldwide with T2D; in 20 years, this number is expected to increase to more than 600 million^2^. The pathophysiological features of T2D include chronic systemic inflammation, oxidative stress, endoplasmic reticulum stress, and apoptosis^2, 3^. Recent studies have reported that autophagic dysfunction is involved in the pathogenesis of T2D, and studies have shown a trend toward increasing insulin resistance and autophagy^2, 4-7^.

Autophagy maintains intracellular homeostasis through protein turnover and is a cell survival mechanism activated by starvation, oxygen-free radicals, and hypoxia^8^. The process of autophagy is initiated through the pathway of nucleation–elongation/enclosure–degradation; autophagosomes are formed, which then fuse with lysosomes, resulting in disintegration of abnormal proteins and cell organelles. Studies by Koga et al.^9^, Kosacka et al.^10^ and Papáčková et al.^11^ showed that an increase in insulin resistance interferes with the fusion of autophagosomes and lysosomes, resulting in defective intracellular autophagy. Liu et al.^12^ studied a mouse model with T2D induced by a high-fat diet, and reported a decrease in the microtubule-associated protein-1 light chain-3 (LC3-II/LC3-I) ratio, a primary marker of autophagic activity, an increase in p62, and a decrease in vacuolar protein sorting 34 (PS34), autophagy-related gene 12 (ATG12), and GABA receptor-associated protein-like 1 gene (GABARAPL1), which are involved in autophagy.

Autophagy in the liver, muscle, heart, pancreas, and skeletal muscle plays an important role in glucose metabolism and utilizes about 80% of the glucose that mediates insulin action^2, 13-16^. Thus, intramuscular insulin resistance is a major cause of glucose intolerance and T2D. Shi et al.^15^ reported reduced insulin resistance associated with autophagic activity in skeletal muscle upon administration of dihydromyricetin, an antioxidant substance, to wild-type experimental animals for 10 weeks. On the basis of their study findings, the researchers applied the same treatment in a T2D experimental animal model induced by a high fat diet, and found that autophagy was induced in skeletal muscle by AMP-activated protein kinase (AMPK), which is the primary mechanism of autophagy. Furthermore, an improvement in T2D with an increase in insulin receptor substrate-1 (IRS-1) has been reported^15^. In addition, Zhou et al.^16^ reported decreased insulin resistance in muscle cells, with improvement in autolysosome activity, which is responsible for disintegration of autogranular bodies, as a result of intermittently administering osteocalcin, an osteogenic hormone, to a T2D mouse model induced by a high fat diet.

Exercise is effective in preventing components of metabolic syndrome, including hypertension, hyperlipidemia, and obesity^17^, and maintains and improve the qualitative and functional status of skeletal muscles. Repetitive muscle contraction through exercise reportedly activates autophagy as part of the mechanism of homeostasis in skeletal muscle^18-20^. According to Lira et al.^21^, the overall improvement in physical status induced by voluntary endurance exercise for 4 weeks is the result of an increase in the basal level of autophagy in skeletal muscle. Grumati et al.^22^ and He et al.^23^ reported an increase in autophagic flux based on an increase in the conversion rate of LC3II/LC3I induced by exercise, and a decrease in p62, an autophagic adapter protein. Furthermore, Masiero et al.^18^ reported a correlation between autophagy and endurance exercise ability, based on the finding that a mouse model lacking the ATG7 gene in skeletal muscle was incapable of normal endurance exercise.

Accordingly, improvement in autophagy through exercise can be an effective noninvasive intervention to prevent progression of T2D. Therefore, the purpose of this study was to investigate changes in blood glucose levels and insulin resistance during an 10 weeks treadmill exercise in a T2D animal model induced by a highfat diet, and to confirm the change of Beclin-1, which is involved in the autophagic initiation process, and LC3-II/I, p62, and LAMP-2, which are involved in the disintegration phase.

## METHODS

### Experimental Animals

The animals used in this experiment were 8-week-old male Sprague Dawley rats (n = 24) from KOATECH, raised in the Korea National Sports University animal laboratory at a temperature of 23±1°C and 40-60% humidity, with a 12-h light-dark cycle. A general diet was given before starting the experimental diet. To obtain similar average weights, rats were divided into ND-SED (Normal Diet-Sedentary, n=8), HF-SED (High-Fat diet-Sedentary, n=8), and HF-TE (High-fat diet + Treadmill Exercise) groups.

ND-SED rats were provided the same feed as used for pre-breeding, while HF-SED and HF-TE rats were provided a diet containing 60% fat. Dietary and water intake were supplied without restriction and body weight was measured twice a week at the same time. The animals were fed a high-fat diet for 35 weeks, followed by 10 weeks of treadmill exercise. All experimental procedures and methods were approved by the Korea National Sports University Animal Experimental Ethics Committee.

### Exercise protocol

Pre-adaptation training (30 min/day, 8 m/min, 5 days/week) was performed on a rodent treadmill (8 Lanes, Daemyung Scientific Co.) by rats given a high-fat diet. The exercise intensity was modified based on the program proposed by Kim et al.^24^ and Choi et al.^25^, and was performed for 10 weeks according to the exercise performance ability of rats with induced obesity. Exercise training was performed at moderate intensity, because the experimental animals were overweight and aging. Therefore, exercise intensity was modified in this study. The exercise protocol is shown in Table 1.

**Table 1.** Treadmill exercise protocol

Intensity	Time	Grade (%)	Frequency (day)
8 m/min	5 min	0	5
11 m/min	5 min		
15 m/min	20 min		
8 m/min	10 min		

### Glucose Tolerance Test

A glucose tolerance test was performed at 2 and 8 weeks of exercise. Rats were fasted for 15 hours before the test; blood was collected from a tail vein and subjected to a pretest (0 min). After glucose (2 mg/kg) was injected, blood was collected at 30 min, 60 min, 90 min, and 120 min. The blood was analyzed using ACCU-CHEK Active (Roche Diagnostics GmbH, Germany).

### HOMA-IR index and Serum biochemical composition

Anesthesia was induced with CO_2_, the abdomen was incised, and 5 ml of blood was collected from the posterior vena cava and centrifuged at 3,000 rpm for 30 min. The supernatant serum was transferred to another tube using a syringe and stored at -80°C until the next analysis. Serum biochemical tests (insulin, triglyceride, total cholesterol, high-density lipoprotein [HDL] cholesterol, low-density lipoprotein [LDL] cholesterol) were sent to the Green Cross Clinical Laboratory for analysis. The Homeostatic Model Assessment of Insulin Resistance (HOMA-IR) score was calculated as HOMA-IR = [fasting insulin (μU/mL) fasting glucose (mmol/L)/22.5] according to the method suggested by Matthews et al.^26^

### Tissue extraction

Exercise and blood glucose tests were conducted for 10 weeks; soleus muscles were excised after anesthesia with CO_2_ gas and stored at -80°C until analysis.

### Sodium dodecyl sulfate polyacrylamide gel electrophoresis

Experiments were conducted using 10% separating gel (3 DW, 30% acrylamide: bisacrylamide, 1.5 M Tris pH 8.8, 10% SDS, TEMED, 10% ammonium persulfate) and 5% stacking gel (3 DW, 30% acrylamide: bisacrylamide, 1 M Tris pH 6.8, 10% SDS, TEMED, 10% ammonium persulfate). A 2× sample loading buffer (60 mM Tris pH 6.8, 25% glycerol, 2% SDS, 14.4 mM 2-mercaptoethanol, 0.1% bromophenol blue) was mixed well at a ratio of 1:1 and protein was denatured by boiling at 95°C. for 10 min and cooled for 10 min on ice, and centrifuged again (14,000 rpm, 10 min). Each sample was combined with a standard marker (Smart Color Protein Marker [EBM-2000; ELPIS]) in a stacking gel prepared in a Mini-Protein II dual-slab apparatus (Bio-Rad, CA, USA), and the total protein amount was adjusted to 40 μg and electrophoresed at 80 volts until it reached bottom.

### Western blot analysis

After immersing the polyvinylidine difluoride membrane in MeOH, the membrane was washed with transfer buffer (190 mM glycine, 50 mM Tris-base, 0.05% SDS, 20% methanol) and transferred at 60 v for 90 min after the Whatman 3M paper was impregnated in the transfer buffer to the Mini trans-bolt cell (Bio-Rad, CA, USA), in sequence. After the membrane was deposited on the rocker platform, the membrane was immersed in a 5% BSA solution (in TBS-T: 10 mM Tris-base pH 8.0, 150 mM NaCl, 0.1% Tween- 300, Jetotech, Korea) for 60 min to block using shaker equipment (SK-300, Jetotech, Korea). The primary antibodies (Table 2) were diluted 1:1,000 in 5% BSA solution and incubated at 4°C for 12 hours. After washing three times for 10 min with 0.1% TBS-T solution, the secondary antibody was diluted 1:5,000 with blocking solution and shaken for 1 h. The membrane was rinsed three times for 10 min with TBS-T solution and added to western blotting luminol reagent solution for 1 min. The obtained membrane was analyzed using an image analysis system (Molecular Imager ChemiDoc XRS System, Bio-Rad, USA), and the amount of protein was calculated using Quantity One 1-D Analysis Software (Bio-Rad, USA).

**Table 2.** List of Primary antibodies.

Antibody	Source	Vender	Catalog No.
α-tubulin	Mouse monoclonal	Santa Cruz	SC-5286
Beclin-1	Rabbit polyclonal	Cell signaling	#3769
p62	Rabbit polyclonal	Cell signaling	#3738
LC3 A/B	Rabbit polyclonal	Cell signaling	#5114
Lamp-2	Goat polyclonal	Santa Cruz	SC-8100

### Statistical analysis

All results obtained in this study were analyzed using IBM SPSS Statistics 22.0 software and the mean ± SD of descriptive statistics for each variable was calculated. One-way analysis of variance (ANOVA) was used to identify differences in variables between the groups. A post-test was conducted using the Bonferroni method to confirm specific differences. The statistical significance level of all tests was set to *α* = .05.

## RESULTS

### Changes in body weight in each group

**Figure 1.**
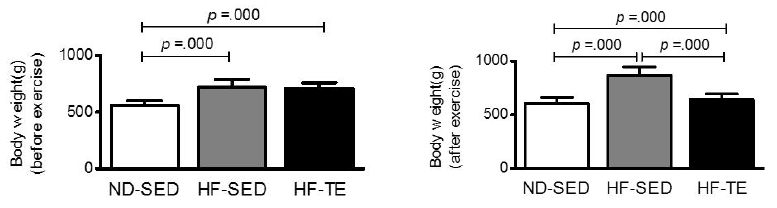
Differences of body weight (before exercise and after exercise) by group. Bonferroni post hoc test after One-way ANOVA. Values are means ± SD of 8 animals/group.

Changes in body weight among groups were measured at the same time twice a week, and means ± SD were used in this study. The rats were fed a high fat diet for 35 weeks and weighed before the start of exercise and after final exercise. The results are shown in Figure 1.

Before starting the treadmill exercise, we compared the average body weights (g) by group: ND-SED (565.62 ± 37.24), HF-SED (720.00 ± 68.60), and HF-TE (711.75 ± 49.36), and found a statistically significant difference [*F* (2, 21) = 21.219, *p* = .000]. Post-verification showed a greater weight in the HF-SED group (*p* = .000) and HF-TE group (*p* = .000) than in the ND-SED group. There was no significant difference between the HFSED and HF-TE groups (*p* = 1.000).

After the end of treadmill exercise, we compared the average body weights (g) by group: ND-SED (607.12 ± 54.18), HF-SED (870.00 ± 77.48), and HF-TE (645.37 ± 47.86), and found a statistically significant difference [*F* (2, 21) = 43.104, *p* = .000]. Post-verification showed a greater weight in the HF-SED group (*p* = .000) and HF-TE group (*p* = .000) than in the ND-SED group. There was a significant decrease in the HF-TE group compared with the HF-SED group (*p* = .000).

### Triglyceride and cholesterol levels in each group

**Figure 2.**
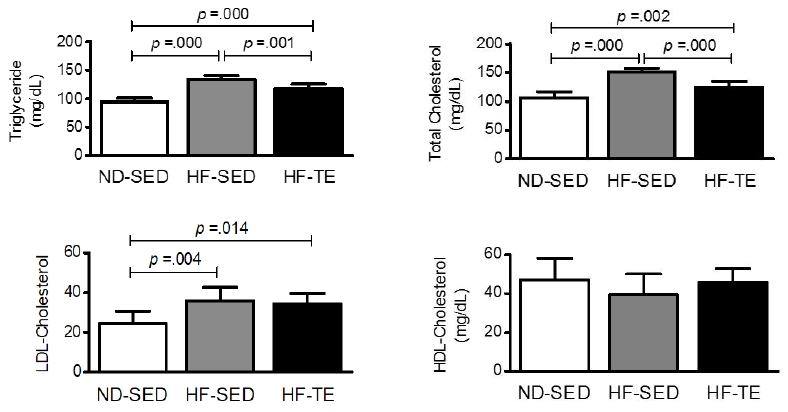
Differences of triglyceride, total cholesterol, LDL-cholesterol, and HDL-cholesterol by group. Bonferroni post hoc test after one-way ANOVA.

Triglyceride, total cholesterol, LDL-cholesterol, and HDL-cholesterol were calculated with a formula. The results are shown in Figure 2.

One-way ANOVA of triglyceride level showed a statistically significant difference between the ND-SED (94.25 ± 7.36), HF-SED (133.75 ± 6.56), and HF-TE (117.00 ± 8.43) groups [*F* (2, 21) = 56.009, *p* = .000]. Post-verification showed that the triglyceride level increased in the HF-SED (*p* = .000) and HF-TE (*p* = .000) groups compared to that in the ND-SED group. However, the triglyceride level was lower in the HF-TE group than in the HF-SED group (*p* = .001).

One-way ANOVA of the total cholesterol level showed a statistically significant difference between groups: ND-SED (106.75 ± 10.43), HF-SED (151.12 ± 6.70), and HF-TE (125.12 ± 10.14) [*F* (2, 21) = 46.470, *p* = .000]. Post-verification found that total cholesterol level increased in HF-SED (*p* = .000) and HF-TE (*p* = .002) groups compared to that in the ND-SED group. However, total cholesterol in the HF-TE group was less than in the HF-SED group (*p* = .001).

One-way ANOVA of the LDL-cholesterol level showed that there was a statistically significant difference between the ND-SED (24.50 ± 6.09), HF-SED (35.87 ± 6.96), and HF-TE (34.25 ± 5.41) groups [*F* (2, 21) = 7.905, *p* = .003]. Post-verification found that LDL-cholesterol level increased in the HF-SED (*p* = .000) and HF-TE (*p* = .014) groups compared to that in the ND-SED group.

ANOVA of the HDL-cholesterol level showed no statistically significant differences between the ND-SED (47.00 ± 11.01), HF-SED (39.50 ± 10.54), and HF-TE (45.87 ± 7.10) groups [*F* (2, 21) = 1.387, *p* = .272].

### Insulin, glucose, and HOMA-IR in each group.

**Figure 3.**
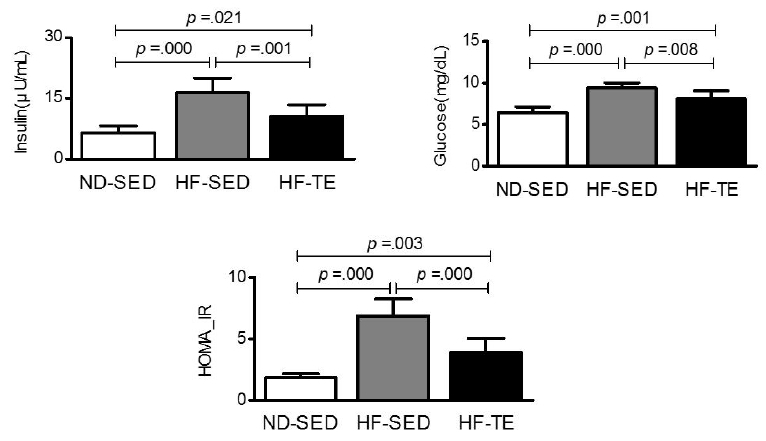
Differences in insulin, glucose, and HOMA-IR by group. Bonferroni post hoc test after One-way ANOVA.

Insulin and glucose levels were determined from blood samples, and the HOMA-IR index was obtained using a formula. The results are shown in Figure 3.

One-way ANOVA of the insulin level showed a statistically significant difference between the ND-SED (6.55 ± 1.75), HF-SED (16.50 ± 3.58), and HF-TE (10.72 ± 2.75) groups [*F* (2, 21) = 25.453, *p* = .000]. Post-verification found that the insulin level increased in the HFSED (*p* = .000) and HF-TE (*p* = .021) groups compared to that in the ND-SED group. However, the level in the HF-TE group was lower than that in the HF-SED group (*p* = .001).

One-way ANOVA of glucose level showed a statistically significant difference between ND-SED (6.43 ± 0.70), HF-SED (9.42 ± 0.59), and HF-TE (8.09 ± 0.98) groups [*F* (2, 21) = 17.895, *p* = .000]. Post-verification found that the glucose level increased in the HF-SED (*p* = .000) and HF-TE (*p* = .001) groups compared to that in the ND-SED group. However, glucose level in the HF-TE group was lower than in the HF-SED group (*p* = .008).

One-way ANOVA of the HOMA-IR index showed a statistically significant difference between the ND-SED (1.83 ± 0.33), HF-SED (6.87 ± 1.39), and HF-TE (3.88 ± 1.19) groups [*F* (2, 21) = 44.174, *p* = .000]. Post-verification found that the index increased in the HF-SED (*p* = .000) and HF-TE (*p* = .003) groups compared to that in the ND-SED group. However, the index in the HF-TE group was lower than in the HF-SED group (*p* = .000).

### Expression level of autophagic factor proteins in soleus muscle

#### Level of Beclin-1 protein in soleus muscle

**Figure 4.**
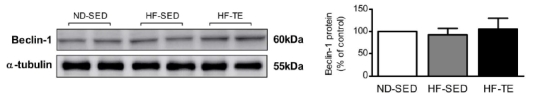
Comparisons of Beclin-1 protein expression level in the soleus muscle by group.

The levels of Beclin-1 protein were as follows: ND-SED (100 ± 0.00%), HF-SED (92.41 ± 14.42%), and HF-TE (105.28 ± 24.38%). There was no statistically significant difference among groups [*F* (2, 15) = .938, *p* = .413] (Figure 4).

In addition, the level in the HF-SED group was decreased compared to the level in the ND-SED group and was increased in the HF-TE group compared to the level in the ND-SED and HF-SED groups, but the differences were not significant.

#### Level of p62 protein in soleus muscle

**Figure 5.**
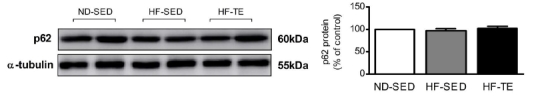
Comparisons of p62 protein expression level in the soleus muscle by group.

The levels of p62 protein were as follows: ND-SED (100 ± 0.00%), HF-SED (96.88 ± 4.56%), and HF-TE (102.10 ± 4.76%). There was no statistically significant difference among groups [*F* (2,15) = 2.839, *p* = .090] (Figure 5).

In addition, the level in the HF-SED group was decreased compared to the level in the ND-SED group and was increased in the HF-TE group compared to the level in the ND-SED and HF-SED groups, but the differences were not significant.

#### LC3-II/LC3-I ratios in soleus muscle

The LC3-II/LC3-I ratios were as follows: ND-SED (100 ± 0.00%), HF-SED (99.84 ± 15.47%), and HF-TE (92.89 ± 12.11%). There was no statistically significant difference among groups [*F* (2,15) = .768, *p* = .482] (Figure 6).

**Figure 6.**
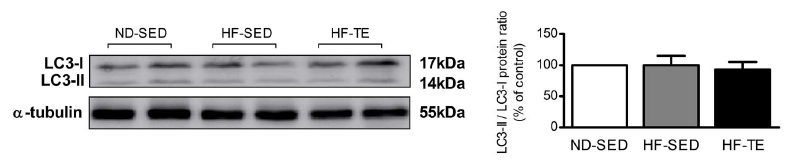
Comparisons of LC3-II / LC-3-I ratio in the soleus muscles by group.

**Figure 6.**
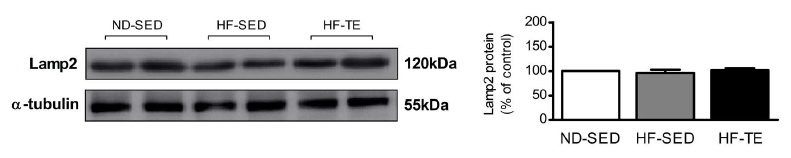
Comparisons of LC3-II / LC-3-I ratio in the soleus muscles by group.

In addition, the ratio in the HF-SED group was decreased compared to the ratio in the ND-SED group and the ratio was decreased in the HF-TE group compared to that in the ND-SED and HF-SED groups, but the differences were not significant.

#### Level of Lamp-2 protein in soleus muscle

The levels of Lamp-2 protein were as follows: ND-SED (100 ± 0.00%), HF-SED (96.24 ± 6.38%), and HF-TE (101.62 ± 4.15%). There was no statistically significant difference among groups [*F* (2,15) = 2.360, *p* = .128].

In addition, the level in the HF-SED group was decreased compared to the level in the ND-SED group, and was increased in the HF-TE group compared to the level in the ND-SED and HF-SED groups, but the differences were not significant.

## DISCUSSION

Reduced activity and excessive dietary intake in association with improved living standards have contributed to increased obesity^27^ and obesity-related disease. Fat accumulation due to ingestion of a high-fat diet has an effect on energy metabolism and function^28^. Excessive fat accumulation is called obesity and causes dyslipidemia. Atherosclerosis and myocardial infarction are associated with obesity and dyslipidemia^29^. With an increase in obesity, the risk of related disease is also increasing. Exercise and dieting can reduce this risk.

Exercise has long been suggested as a good way to treat and prevent obesity. In particular, endurance exercise is effective in reducing total body fat, reducing visceral fat, and improving insulin resistance by enhancing glucose metabolism^30^. According to a study by Delghingaro-Augusto et al.^31^, rats with obesity induced by a high-fat diet tended to have decreased body weight and body fat after spontaneous wheel exercise for 6 weeks. In the case of autophagy, Levine et al.^32^ reported that exercise stimulates autoregulation and alleviates neurodegenerative diseases such as Alzheimer's disease and diabetes.

Autophagy is very important because it affects the removal of abnormal proteins as well as infectious agents such as bacteria and viruses^33^. Therefore, the purpose of this study was to investigate the general characteristics, changes in blood markers, and the expression level of proteins related to autophagy in the soleus muscle after 10 weeks of endurance exercise in rats with obesity induced by a 60% high-fat diet.

Weight was increased in both HF-SED and HF-TE groups in comparison with the ND-SED group, and showed a noticeable decrease in the HF-TE group. In addition, triglyceride, total cholesterol, LDL-cholesterol, insulin, glucose, and HOMA-IR values increased in the HF-SED group. In other words, increased body weight was reduced through endurance exercise despite a high-fat diet. According to McTiernan et al.^34^, endurance exercise activates fat-splitting enzymes, resulting in a decrease in body weight, abdominal fat percentage, and body fat. Additionally, western blotting was conducted to analyze autophagic flux and how a high-fat diet affects the process of autophagy. Tissue in the soleus muscle was used for analysis. Autophagic flux refers to a series of processes in which degradation occurs from the initiation of autophagy^35^.

Beclin-1 is a protein that affects the initiation stage of autophagosome formation. There was no significant difference in the level of Beclin-1 expression in the ND-SED, HF-SED, and HF-TE groups. These results suggest that high-fat diets did not affect the initiation of autophagy.

The p62 (sequestosome 1) protein plays a role as an adapter to promote autophagy through association with ubiquitinated proteins^36, 37^. Thus, p62 is increased when the autophagy process becomes active^38^. However, in this study, the expression of p62 was expected to increase in the HF-SED group with a high-fat diet, but the difference was not statistically significant. Previous studies have shown that p62 increased in the fasting group, but not in the fasting group. This is consistent with the findings in this study^39^.

The LC3 protein is a key marker of autophagy, which is involved in the formation of autophagosomes by binding to phosphatidylethanolamine^40^. Penfei et al.13 showed that the levels of streptozotocin-induced autophagy-related factors such as Beclin-1 and LC3 in diabetic rats did not change statistically when compared to those in the ND-SED group. These results show that there is no change in the process of autophagy.

Lysosome-associated membrane protein-2 (Lamp-2) protein is important in the fusion of autophagosomes and lysosomes and requires enzymes such as cathepsins B, D, and L, which are lysosome resolvents. The expression level of Lamp-2 protein was not significantly different in this study. In other words, this study showed that a high-fat diet and exercise did not affect the autophagy process.

The soleus muscle was exercised on the treadmill, and was analyzed in this study. However, there was no significant difference in the results of western blot analysis. For this reason, Jamart et al.^39^ reported that LC3- II b expression was increased when fasting and exercise were performed simultaneously. The authors reported that autophagy was not activated because autophagy had already adapted to hyperglycemia in the muscles of the high-fat diet group, with differences in muscle mass in each group7. Therefore, chronic hyperglycemia in which fat accumulates due to long-term high-fat diets adversely affects not only skeletal muscle but also autophagy in the liver and adipose tissue.

Although the reason is unclear, the method and intensity setting of the exercise performed in this study may be one of causes. Treadmill exercise can be an effective way to reduce weight, but may be less effective than resistance exercise to improve muscle performance. There was no difference in soleus muscle weight between the groups. In addition, we tried to apply moderate-intensity exercise as suggested by Kim et al. (2003)^24^ and Jee et al. (2008)^25^, but exercise was not performed normally because the HF-SED group was overweight. Therefore, inevitably, low-intensity exercise was performed. For this reason, exercise may not have had much effect on the process of autophagy.

In addition, the muscle fiber distribution in the soleus muscle might have affected the process of autophagy. Penfei et al.^13^ reported that the gastrocnemius muscle responds more sensitively to autophagy than the soleus muscle. This may be due to the control of protein synthesis and differences between slow-twitch and fasttwitch muscle fibers. In other words, the gastrocnemius muscle compared with the soleus muscle is more susceptible to external changes such as starvation, resulting in protein turnover^41^. Autophagy tends to be activated in the starvation state. However, a hyperlipidemic state, in which glucose is elevated by a long-term high-fat diet, results in an excess of nutrients rather than a starvation state.

This study showed statistically significant increases in body weight, blood chemistry levels (total cholesterol, LDL-cholesterol, HDL-cholesterol, glucose), and the HOMA-IR index in the high-fat diet group. However, endurance exercise using a treadmill led to improved body weight, insulin resistance, and blood lipid levels. Western blotting was performed to examine flux in the soleus, but there was no significant difference between the groups in any part of the autophagic process. These results indicate that aerobic exercise can be effective in the treatment of obesity, but does not seem to affect autophagy. However, studies should be performed based on the characteristics of different muscle fibers.

## CONCLUSION

This study investigated the effects of treadmill exercise on body weight and blood biochemical indicators, and analyzed HOMA-IR indices and autophagy-related factors in obese rats. Treadmill exercise led to improvement in body weight, body fat, and biochemical indicators of obesity and Type 2 diabetes, but did not affect autophagy in the soleus muscle.
